# A Contrast Augmentation Approach to Improve Multi-Scanner Generalization in MRI

**DOI:** 10.3389/fnins.2021.708196

**Published:** 2021-08-31

**Authors:** Maria Ines Meyer, Ezequiel de la Rosa, Nuno Pedrosa de Barros, Roberto Paolella, Koen Van Leemput, Diana M. Sima

**Affiliations:** ^1^Department of Health Technology, Technical University of Denmark, Lyngby, Denmark; ^2^Icometrix, Leuven, Belgium; ^3^Department of Computer Science, Technical University of Munich, Munich, Germany; ^4^Imec Vision Lab, University of Antwerp, Antwerp, Belgium; ^5^Martinos Center for Biomedical Imaging, Massachusetts General Hospital and Harvard Medical School, Boston, MA, United States

**Keywords:** multi-scanner, magnetic resonance imaging, segmentation, data augmentation, gaussian mixture models

## Abstract

Most data-driven methods are very susceptible to data variability. This problem is particularly apparent when applying Deep Learning (DL) to brain Magnetic Resonance Imaging (MRI), where intensities and contrasts vary due to acquisition protocol, scanner- and center-specific factors. Most publicly available brain MRI datasets originate from the same center and are homogeneous in terms of scanner and used protocol. As such, devising robust methods that generalize to multi-scanner and multi-center data is crucial for transferring these techniques into clinical practice. We propose a novel data augmentation approach based on Gaussian Mixture Models (GMM-DA) with the goal of increasing the variability of a given dataset in terms of intensities and contrasts. The approach allows to augment the training dataset such that the variability in the training set compares to what is seen in real world clinical data, while preserving anatomical information. We compare the performance of a state-of-the-art U-Net model trained for segmenting brain structures with and without the addition of GMM-DA. The models are trained and evaluated on single- and multi-scanner datasets. Additionally, we verify the consistency of test-retest results on same-patient images (same and different scanners). Finally, we investigate how the presence of bias field influences the performance of a model trained with GMM-DA. We found that the addition of the GMM-DA improves the generalization capability of the DL model to other scanners not present in the training data, even when the train set is already multi-scanner. Besides, the consistency between same-patient segmentation predictions is improved, both for same-scanner and different-scanner repetitions. We conclude that GMM-DA could increase the transferability of DL models into clinical scenarios.

## 1. Introduction

The segmentation of different brain structures from Magnetic Resonance Imaging (MRI) is an important problem in the field of neuroimaging. Obtaining precise and consistent delineations is crucial in the diagnosis, follow-up and treatment of neurological disorders. Important examples are the monitoring of the progression of Multiple Sclerosis (MS) or dementia, both connected to accentuated neurodegeneration (Giorgio and De Stefano, 2013). In recent years, convolutional neural networks (CNN) have become an increasingly popular segmentation approach, but the fact that these models are extremely sensitive to data variability has hindered their large scale adoption in clinical and research settings. Specifically, these algorithms remain sensitive to factors such as hardware and acquisition settings, which can be especially problematic when integrating data from different cohorts (Mårtensson et al., 2020). For these models to generalize to data collected using new or unseen scanners, large multi-center and multi-scanner datasets are necessary at the training stage. Nevertheless, collecting such data is not trivial and most available datasets are homogeneous in terms of scanner types and acquisition protocols.

### 1.1. Related Work

The above mentioned problem is often termed as the *scanner bias* problem. A popular way to deal with it in large clinical trials is through approaches based on statistical *harmonization*. In most cases the focus is on removing the scanner bias from the volumetric measurements based on scanner- or center-information (Fortin et al., 2018; Garcia-Dias et al., 2020). At the image level, it is common to use the standardization of the MRI intensity scale to reduce scanner sensitivity (Wang et al., 1998; Nyúl and Udupa, 1999; Shinohara et al., 2014), which has been previously shown to improve the outcome of computer vision tasks like segmentation (Zhuge and Udupa, 2009) and registration (Bagci et al., 2010). Recently, some works have attempted to use Deep Learning (DL) methods to modify the analyzed images such that they appear to have been acquired under similar settings (Dewey et al., 2019; Zhao et al., 2019b). However, harmonization methods have the undesirable property that the results will always be bound by the least informative scanner in the dataset, as shown in Moyer and Golland (2021), while standardization methods are not able to remove residual across-subject variability (Shinohara et al., 2014; Fortin et al., 2016; Wrobel et al., 2020). Additionally, many of these approaches require retraining and updating of the models when including new data from unseen scanners or centers.

In order to avoid these unwanted effects, it is interesting to tackle the problem from a generalization perspective, by improving the performance and reproducibility of the methods of interest (often segmentation of brain tissues or lesions). When considering DL methods in particular, a common approach is to increase the variability in the data by applying well designed data augmentation (DA). The idea behind DA is simple: by applying transformations to the labeled data it is possible to artificially increase the training set, which implicitly regularizes the trained CNN. The most common DA strategies explore transformations of the original data, mostly based on the application of operations such as elastic distortions (Simard et al., 2003), linear geometric transformations such as translations and rotations, color transformations (mostly by altering the intensities of the RGB channels in 2D images) (Krizhevsky et al., 2012) or noise injection (Sietsma and Dow, 1991).

In the medical imaging field, DA is especially important since annotated datasets are typically small. Although simple transformations such as the ones described above can alleviate overfitting and improve performance on the test sets in medical applications (Milletari et al., 2016), they do not take into account the high variability in terms of contrast found in MRI. Some works have attempted to overcome this limitation by generating completely synthetic images using generative adversarial networks, as is the case in Shin et al. (2018). Nonetheless, there is still a long way to go until these images can be used effectively. Other more promising approaches start from existing images and alter them in such a way that new sequences or contrasts are simulated. One relevant example is described in Jog et al. (2019), where a CNN-based algorithm resilient to variations in the input acquisition is presented. To achieve this, approximate forward models of different MRI pulse sequences are built. This way, synthetic versions of the training images are generated such that they appear to have been acquired using different sequences. The method has the disadvantage that it is complex, slow and it requires nuclear magnetic resonance parameter maps of the training images, which are often unavailable. Zhao et al. (2019a) proposed to learn a model of transformations from an atlas to images in a dataset and to use this model along with a single labeled example to synthesize additional labeled examples with variable appearance and spacial deformations. More recently, Billot et al. (2020) presented a contrast-agnostic brain segmentation method, again based on generating synthetic images. The method uses only a segmentation map to generate new images with varying, sometimes even unrealistic, contrasts. The generated images have random appearance, deformation, noise, and bias field. With this type of extreme augmentation, it is possible to obtain a final model that is not biased toward any specific MRI contrast and that achieves good performance on unpreprocessed brain scans of any contrast. Although this method is very promising, by design it is limited to segmentation applications and nuanced variations in the individual images are lost.

### 1.2. Our Contribution

In the present work we propose a novel intensity-based DA strategy with the main goal of reducing the scanner bias of models trained on data with low protocol-, scanner- or center- variability. Although scanner factors cause variations to other image characteristics (e.g., noise, artifacts, geometric distortions), we have previously found a clear relationship between tissue contrast and volume measurements (Meyer et al., 2019). As such, we hypothesize that augmenting the tissue intensity variability will have a positive effect in the model generalization to new, unseen scanners or center-specific acquisition configurations. The method is based on the Gaussian Mixture Model (GMM) framework: we estimate the individual tissue components of an MRI image and randomly modify them, while preserving structural information. As a result the contrast between different tissues varies, in a similar way to what happens when different scanners or sequences are used during acquisition. We validate the approach in the task of brain structure segmentation. Unlike currently existing methods, the proposed approach does not depend on any existent segmentations or parameter maps; it is simple and fast; it can be used on-the-fly during training; and it is not necessarily limited to segmentation applications.

This work extends the preliminary research presented in Meyer et al. (2021). We previously compared the performance of the same CNN-based model trained under three different settings: (i) single-scanner data, (ii) single-scanner data with the addition of our DA method, and (iii) multi-scanner data. We now additionally investigate the effect of adding the DA method to multi-scanner data and evaluate the reproducibility and consistency of the models on a *test-retest* dataset containing same-patient repetitions in the same and different scanners. Finally, we investigate the effect of the presence of bias field on the training images. Overall we observe a clear improvement in generalization to unseen scanner types when adding the proposed method to the training pipeline, not only when the original training dataset is homogeneous, but also in the case when a large, heterogeneous dataset is used as training set.

## 2. Gaussian Mixture Model-Based Intensity Transformation

**Figure 1:**
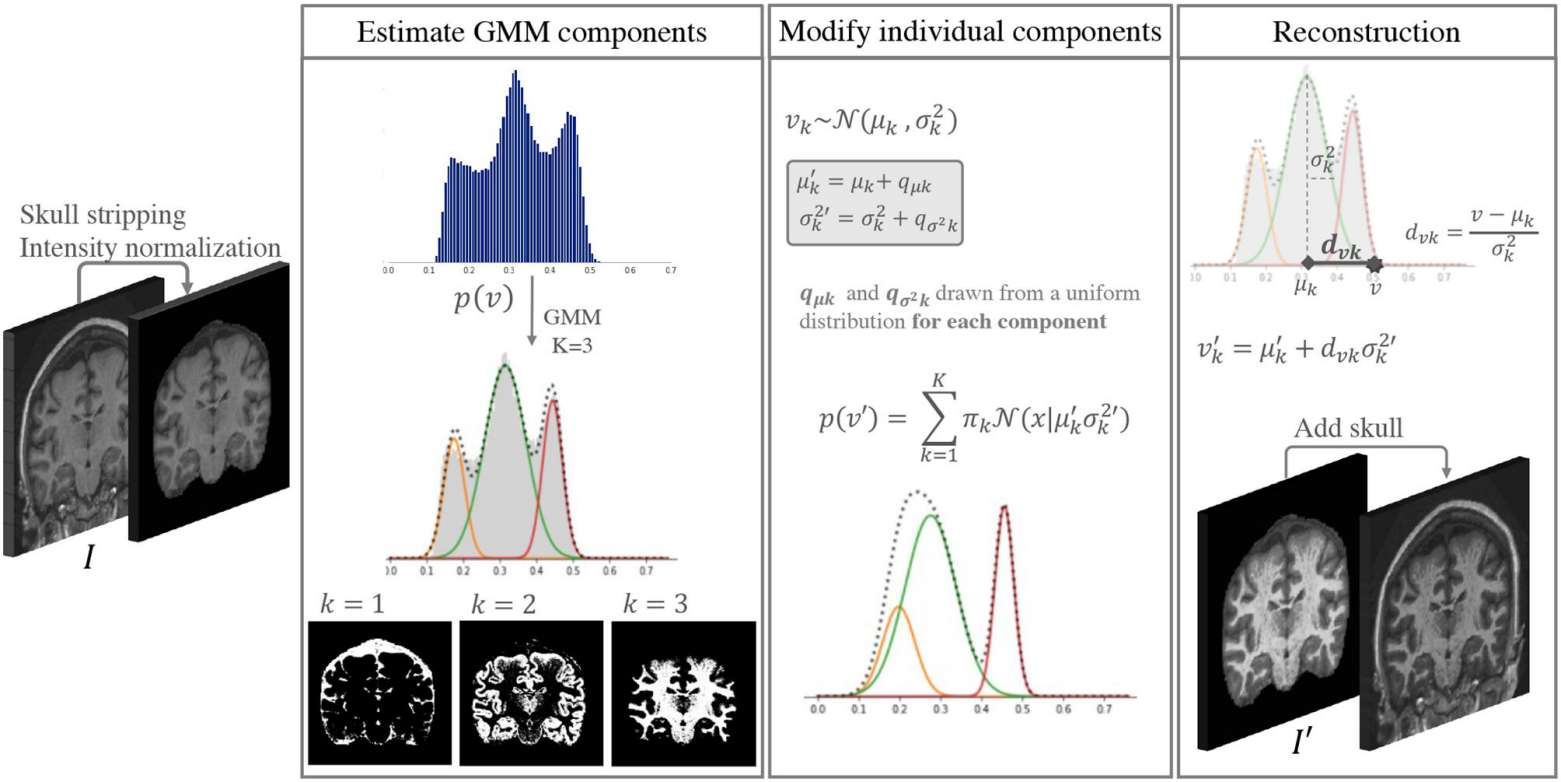
Diagram of the main steps in the proposed DA method. This augmentation is performed only while training the segmentation network.

The idea behind the proposed approach is to increase the intensity and contrast variability of images in datasets with low scanner and center acquisition diversity, such that it becomes representative of what is found in large multi-scanner and multi-center cohorts. This DA method is applied during the training phase of a DL network of choice, and is not necessary at inference. Figure 1 shows a depiction of the method. An implementation is available at https://github.com/icometrix/gmm-augmentation.

### 2.1. The Gaussian Mixture Model Framework

It is well documented that in a skull-stripped T1w brain MRI without contrast injection, characteristic peaks in the histogram correspond to different tissues, i.e., CSF has the lowest intensity, followed by GM and WM. This has been explored by several segmentation methods based on Gaussian Mixture Models (GMM) (Van Leemput et al., 1999; Ashburner and Friston, 2005). GMM is a type of probabilistic model that assumes that data can be modeled as a superposition of *K* Gaussians. Within this framework, if we have a set of observations {*v*_1_, …*v*_*N*_}, corresponding to the intensities *v* of each voxel *n* ∈ *N* in an image *I*, we can model each observation in the data using a mixture of Gaussians, such that:

(1) $$\begin{array}{l} p\left(v_{n}\right)=\overset{K}{\underset{k=1}{\sum }}\pi_{k}N\left(v_{n}|\mu_{k},\sigma_{k}^{2}\right). \end{array}$$

Each $N\left(\mu_{k},\sigma_{k}^{2}\right)$ is a *component* of the mixture, with its own mean μ_*k*_ and variance $\sigma_{k}^{2}$, and π_*k*_ are the mixing coefficients. For simplicity we hide the subscript *n* when referring to the intensity of a given voxel: *v*_*n*_ is represented as *v* from here on.

We start by selecting *K* = 3 Gaussian components for the GMM, where each component roughly corresponds to the CSF, GM, and WM classes. The parameters are initialized and updated iteratively using the Expectation Maximization (EM) (Dempster et al., 1977) algorithm implemented in the scikit-learn package for Python (Pedregosa et al., 2011) with default parameters.

Once we estimate the parameters for each component *k*, we can use Bayes' rule to compute the probability of each class label *C*, such that:

(2) $$p(C=k|v)=\frac{\pi_{k}N(v|\mu_{k},\sigma_{k}^{2})}{\sum_{k^{\prime }=1}^{K}\pi_{k^{\prime }}N(v|\mu_{k^{\prime }},\sigma_{k^{\prime }}^{2})}.$$

### 2.2. Altering the Components of the GMM

If we modify the individual components of a 3-component GMM we can modify images in the training data by changing their GMM probability distributions while preserving the inherent image characteristics. We can create a new intensity distribution for each of the tissues by generating new parameters $\mu_{k}\to \mu_{k}^{\prime }$ and $\sigma_{k}^{2}\to \sigma_{k}^{2\prime }$ for each of the components in an individual skull stripped image. To do this we:

a) sample individual variation terms *q*_μ_*k*__ and $q_{\sigma_{k}^{2}}$ for each component from a uniform distribution,
b) add these values to the original parameters, such that $\mu_{k}^{\prime }=\mu_{k}+q_{\mu_{k}}$ and $\sigma_{k}^{2\prime }=\sigma_{k}^{2}+q_{\sigma_{k}^{2}}$.

To define the range of the uniform distributions we use to sample the variation terms *q*_μ_*k*__ and $q_{\sigma_{k}^{2}}$, we start by estimating the range of typical variation for each component from a large multi-scanner collection of patient data (dataset C in section 3). To do this, all images are first skull stripped, intensities are clipped at percentiles 1 and 99 to remove extreme values, and normalized to the range [0, 1]. Then we fit a 3-component GMM to each image in the dataset using the same procedure as described above. We extract the mean μ_*k*_ and variance $\sigma_{k}^{2}$ values of each component. We then use the standard deviation (*s*(·)) of the estimated parameters to define the range of variability we allow. *q*_μ_*k*__ and $q_{\sigma_{k}^{2}}$ are sampled for each component from the uniform distributions *U*(−*s*(μ_*k*_), *s*(μ_*k*_)) and $U\left(-s\left(\sigma_{k}^{2}\right),s\left(\sigma_{k}^{2}\right)\right)$, respectively.

**Figure 2:**
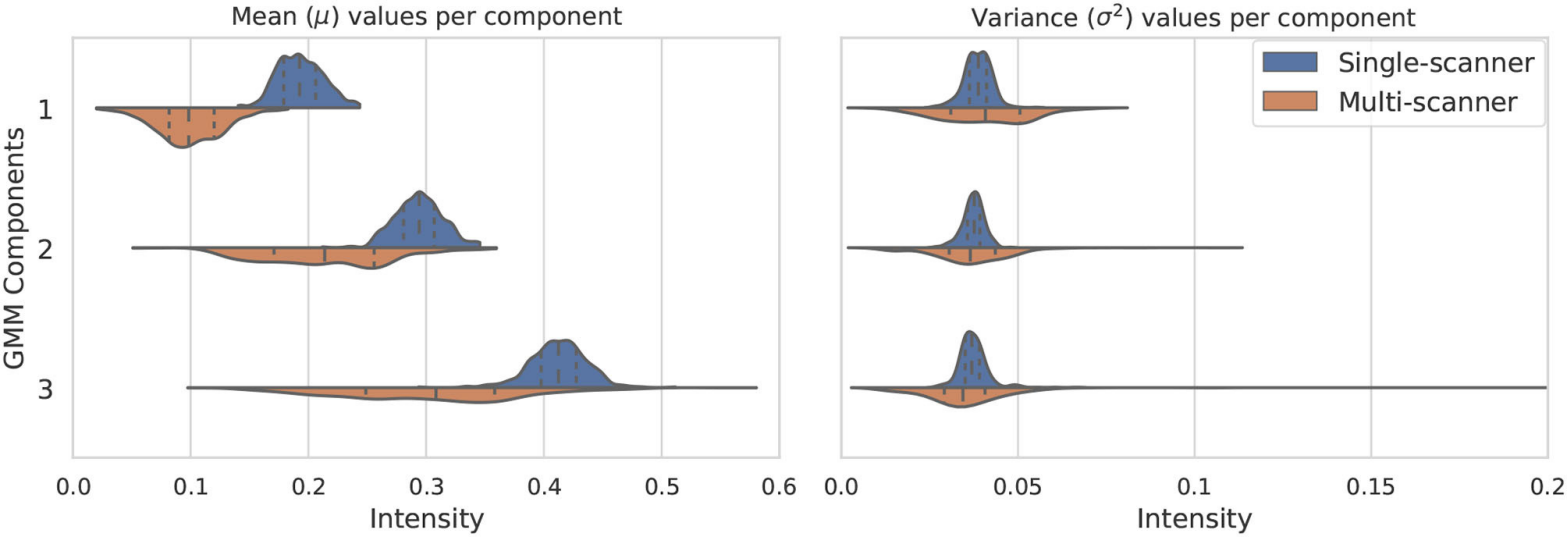
Variation of the three considered GMM components in terms of mean μ_*k*_ (left) and variance $\sigma_{k}^{2}$ (right). The components vary much more in a multi-scanner dataset than in a single-scanner setting.

The distribution of the estimated parameters μ_*k*_ and $\sigma_{k}^{2}$ is depicted in Figure 2. In this figure we illustrate how the variability of the estimated parameters in a multi-scanner and multi-center setting is larger than that of a homogeneous dataset (same center, same scanner, same acquisition protocol) (dataset A in section 3). Besides differences in hardware, acquisitions in different centers tend to not be perfectly harmonized, which causes variations in contrast of the images. This is one of the many factors that contribute to the increased variability of the estimated parameters, and can be addressed by the proposed approach. For the multi-center data, mean and variance values for the 3 components have approximate standard deviations of *s*(μ) = {3, 6, 8}×10^−2^ and *s*(σ^2^) = {1, 1, 3}×10^−3^, respectively.

The choice of a uniform distribution for sampling the new variation terms implies that any random combination of tissue intensities can be generated. We could restrict this to more probable distributions by selecting a normal distribution. However, since exposing networks to extreme but anatomically plausible augmentation can be beneficial for learning (Billot et al., 2020), we decided to allow the possibility for some unrealistic combinations to arise.

### 2.3. Reconstruction

Once the new parameters have been defined, we could think that a logical next step would be to generate a new histogram of intensities by mixing the new Gaussian distributions and using histogram matching (Wang et al., 1998) techniques to generate a new image *I*′. However, doing this would not guarantee that structural information is preserved (e.g., two components could overlap or even shift order, and voxels from one tissue would be wrongly assigned to another class). To avoid this we describe the intensity *v* of some voxel *n* ∈ *N* in terms of the distance from the mean of the component measured with the Mahalanobis distance *d*_*vk*_ = (*v* − μ_*k*_)/σ_*k*_. This implies that if we know the values of μ_*k*_ and $\sigma_{k}^{2}$ we can find the updated value of *v* → *v*′ for each component *k* by preserving the distance *d*_*vk*_:

(3) $$\begin{array}{l} v_{k}^{\prime }=\mu_{k}^{\prime }+d_{vk}\sigma_{k}^{\prime }. \end{array}$$

Finally, we can compute the new intensity *v*′ for a voxel *n* by leveraging each component by the initial probability that this voxel belonged to a certain class *p*(*C* = *k*|*v*), such that

(4) $$\begin{array}{l} v^{\prime }=\overset{N}{\underset{k=1}{\sum }}p\left(C=k|v\right)v_{k}^{\prime }. \end{array}$$

This guarantees that the voxels that have a high probability of belonging to a certain class will represent the same class, while allowing for nuanced variations at the borders between different tissues.

## 3. Datasets and Experimental Setup

From here onwards, the method will be referred to as *GMM-DA*, for simplicity. In order to validate the GMM-DA method, we investigate the added value of the described method on the task of brain structure segmentation using a well described type of CNN architecture. We train the same network on two different datasets: a collection of single-scanner data from healthy subjects, and a multi-scanner and multi-center collection of patient data. We compare the performance of the models trained with and without the addition of the GMM-DA strategy. The different models are evaluated on manual segmentations and on test-retest data. The available datasets and the different experiments are described in the following sections.

### 3.1. Available Datasets

#### A) OASIS

Contains T1w MRI scans from 416 subjects (age: [18, 96] years) (the OASIS-1 cohort) (Marcus et al., 2007). Only 280 of the 316 healthy subjects were considered (see dataset B). The data was randomly split into train/validation/test sets [*n* = 179(64%)/45(16%)/56(20%)]. Although the data is heterogeneous from a population point of view, it is extremely homogeneous in terms of protocol and scanner. All images were acquired on a 1.5T Siemens Vision scanner, using the MP-RAGE sequence with constant repetition time (TR) and echo time (TE) (TR: 9.7 ms; TE: 4.0 ms). Slice thickness is also constant (1.25 mm).

#### B) MICCAI 2012

Contains 35 T1w scans from healthy subjects. The original MRI scans are from OASIS, but this dataset contains manual labels of brain structures. These data were provided for use in the MICCAI 2012 Grand Challenge and Workshop on Multi-Atlas Labeling (Landman and Warfield, 2012). All the images in this dataset were removed from OASIS prior to splitting the data into the different training and test sets, to avoid overlap. We exclude 5 scans from repeated subjects and use the remaining 30 for evaluating the methods on the manual labels.

#### C) MS Dataset

This is a collection of multi-center T1w MRI scans from 421 individual Multiple Sclerosis (MS) patients. It contains a lot of variability both at the population level and in terms of scanner- and center- or acquisition-specific factors, i.e., age ([16, 81] years), sex (M/F ~ 33%/67%), slice thickness in T1 ([0.4, 1.5] mm), magnetic field strength (1.5T/3T ~ 43%/57%), scanner manufacturer (Philips, GE, Siemens and Hitachi), scanner model (29 devices) and acquisition sequence (TR: [4.9, 5000] ms; TE: [1.9, 8.0] ms). This dataset, which we term *heterogeneous*, was used to estimate the range of typical variation of the GMM components for the different tissues, as described in section 2.2. Additionally, we used this data to generate an *independent test set*, containing 92 images from 10 different scanner models. For an additional experiment we pooled a train/validation set of 251/44 images, ensuring that any scanner models present in the pre-selected test set or in OASIS were not included.

#### D) Test-Retest Dataset

Contains T1w MRI scans from 10 MS patients. Each patient was scanned twice (with re-positioning) on three different 3T scanner types with different acquisition sequences: (i) Philips Achieva: 3D T1-weighted FSPGR sequence (TR 4.93 ms); (ii) Siemens Skyra: 3D T1-weighted MP-RAGE sequence (TR 2300 ms, TE 2.29 ms); (iii) GE Discovery MR450w: 3D T1-weighted FSPGR sequence (TR 7.32 ms, TE 3.14 ms). Further details regarding this data can be found in Jain et al. (2015). This dataset allows the models to be tested for consistency, both in an *intra-scanner* setting as well as in an *inter-scanner* setting.

### 3.2. Data Pre-processing

All images were normalized using a modified z-score function robust against outliers, where the median of the distribution was preferred over of the mean, and the standard deviation of the distribution was computed within percentiles 10 and 90. Additionally, images were bias-field corrected using the N4 inhomogeneity correction algorithm as implemented in the Advanced Normalization Tools (ANTs) toolkit (Tustison et al., 2010) and linearly registered to MNI space using the tools implemented in NiftyReg (Ourselin et al., 2001).

### 3.3. Experimental Setup

We trained a CNN to segment White Matter (WM), Gray Matter (GM), Cerebro-Spinal Fluid (CSF), Lateral Ventricles (LV), Thalamus (Tha), Hippocampus (HC), Caudate Nucleus (CdN), Putamen (Pu) and Globus Palidus (GP). Due to scarcity of manual delineations, we train and evaluate the CNN models using brain substructure delineations obtained with ico**brain** (Jain et al., 2015; Struyfs et al., 2020), a clinically available and FDA-approved Software.

### 3.4. Model Architecture

For the segmentation task we use a 3D UNet architecture (Çiçek et al., 2016) with a few adaptations, namely:

- Weight normalization layers (Salimans and Kingma, 2016) are added after each convolutional operation instead of batch normalization;
- LeakyReLU (Maas et al., 2013) is used as the main activation function;
- Strided convolutions are used instead of max pooling.

The models are trained using a combination of the soft-dice loss ($L_{Dice}$) and the weighted categorical cross-entropy loss ($L_{\text{w}CE}$), as suggested in Isensee et al. (2021):

(5) $$\begin{array}{l} L_{total}=L_{\text{w}CE}+L_{Dice}. \end{array}$$

Considering that *y*_*n*_ ∈ {0, 1} is the one-hot-encoded label of the *n*^*th*^ voxel in the model's input and ŷ_*n*_ ∈ [0, 1] is the prediction output of the model for the same voxel, the soft-Dice loss is an extension to *K* classes of the popular Dice loss presented, as presented in Sudre et al. (2017):

(6) $$L_{Dice}=1-2\frac{\sum_{k=1}^{K}\sum_{n}{\hat{y}}_{nk}y_{nk}}{\sum_{k=1}^{K}\sum_{n}{\hat{y}}_{nk}+y_{nk}}.$$

To deal with the accentuated class imbalance of this problem we use the weighted categorical cross-entropy loss similarly to what was described in Ronneberger et al. (2015). This loss function can be expressed as:

(7) $$\begin{array}{l} L_{wCE}=-\frac{1}{N}\overset{N}{\underset{n=1}{\sum }}\overset{K}{\underset{k=1}{\sum }}w_{nk}y_{nk}\log{ŷ}_{nk}, \end{array}$$

where *w*_*nk*_ is the weighting factor for the *n*-th voxel belonging to the *k*-class in the training set. These weights allow to compensate the scarcity of voxels from some of the classes.

The network takes as input patches of size 128 × 128 × 128 and outputs probability maps of size 88 × 88 × 88. Kernel size is 3 × 3 × 3 and initial number of filters 16 (raised to the power of 2 at each layer in the encoder path). The model is implemented using Tensorflow 2.0 and trained until convergence using mini-batch stochastic gradient descent (Adam optimizer) with initial learning rate λ = 0.001 on a machine equipped with a Tesla K80 Nvidia GPU (12 GB dedicated).

### 3.5. Experiments

To validate the approach we compare the performance of models trained with and without the addition of the GMM-DA strategy. First, we evaluate how a model trained on single scanner data generalizes to an unseen multi-scanner dataset (train on the OASIS training set, and evaluate on the OASIS test set and the MS dataset test set). This is the key experiment in the results, since we are particularly interested in evaluating the increase in generalizability of the CNN to multi-scanner and multi-center data after adding the augmentation step. Although we acknowledge the presence of white matter lesions in the images from the MS dataset, we decide not to deal with them explicitly in this context. Secondly, in order to evaluate how the same network performs on unseen scanners and centers when trained on heterogeneous data, we train the same models on the MS dataset described in section 3. We additionally investigate if the addition of GMM-DA in this setting is still beneficial. We proceed to compare these four approaches on manual labels and on the test-retest dataset. Finally, we evaluate how the presence of bias field (BF) on the training images impacts the performance of the GMM-DA. To this end, we train the same models on the MS dataset images, this time without the bias field correction step.

We train and evaluate a total of six models. The models are named according to the architecture (CNN), training data (OASIS or MS), presence of bias field (BF) on the training images and addition of the data augmentation (DA) step. As such, a model trained on the MS dataset, on data with bias field and to which GMM-DA was applied is termed CNN_MS-BF-DA_. The investigated models and a description of the data where they were trained (T) or evaluated (E) are summarized in Table 1.

**Table 1:** Summary of the trained models.

	**Training/Testing datasets**		**Testing datasets**	
**Model types**	**OASIS**	**MS dataset**	**MICCAI 2012**	**Test-retest**
CNN_OASIS_	T, E	E	E	E
CNN_OASIS-DA_	T, E	E	E	E
CNN_MS_	E	T, E	E	E
CNN_MS-DA_	E	T, E	E	E
CNN_MS-BF_	-	T, E	-	-
CNN_MS-BF-DA_	-	T, E	-	-

*T, trained; E, evaluated*.

### 3.6. Performance Metrics

**Figure 3:**
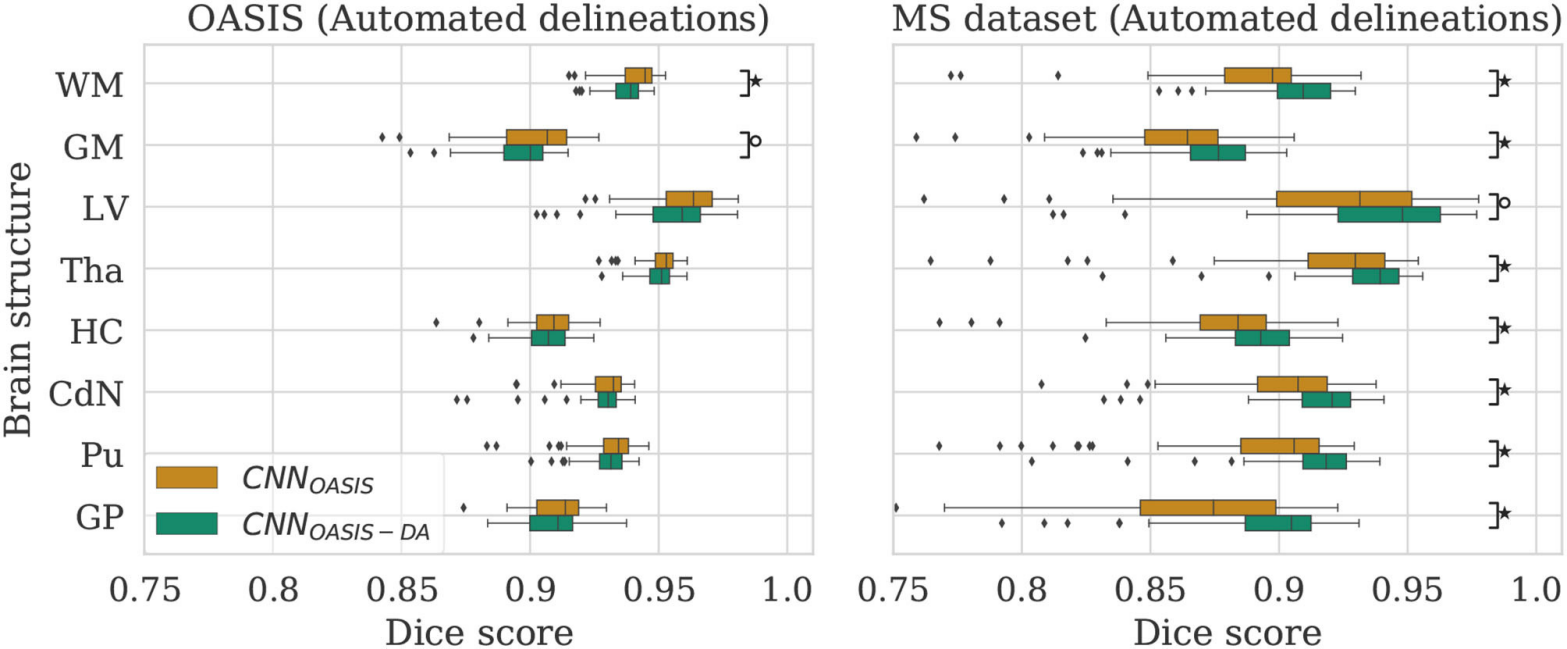
Dice scores for the CNN_OASIS_ and CNN_OASIS-DA_ models on the OASIS (left) and MS dataset (right) test sets. Marks indicate that there is a significant difference between the two models (Wilcoxon, °*p* < 0.05, ^⋆^*p* < 5×10^−4^).

Dice scores (*DC*), sensitivity (*Se*) and precision (*Pr*) are reported (complete *Se* and *Pr* results are given in the Supplementary Material). *DC* values are compared using Wilcoxon paired rank-sum and Levene tests to evaluate the null hypotheses *H*_0_ that the results from the different models have equal median and variance values, respectively. These tests were selected given the presence of outliers and deviations from normality in the distributions (see Figure 3). When evaluating the reproducibility of the methods, absolute volume differences between acquisitions from the same patient are reported. Results are summarized in terms of median (*P*50) and percentile 10 (*P*10) or 90 (*P*90), where relevant.

## 4. Experiments and Results

### 4.1. GMM Augmentation of a Homogeneous Dataset

To evaluate the influence of the addition of GMM augmentation when training on a homogeneous dataset (OASIS), we test CNN_OASIS_ and CNN_OASIS-DA_ on the two cross sectional datasets with automated delineations (test sets of OASIS and MS dataset). This will allow us to determine: (i) if applying GMM-DA decreases the performance on data similar to the training set in comparison to the base model, and (ii) how the models perform in a multi-scanner setting. The results in terms of Dice scores are summarized in Figure 3 and Table 2. The corresponding *Se* and *Pr* results can be found in the Supplementary Table 1.

**Table 2:** Summary of the Dice score (DC) performance of models trained on the OASIS data (CNN_OASIS_ and CNN_OASIS-DA_) and tested on the OASIS and MS dataset test sets.

	**OASIS-test set**		**MS dataset-test set**	
**Tissues**	**CNN_OASIS_**	**CNN_OASIS-DA_**	**CNN_OASIS_**	**CNN_OASIS-DA_**
WM	**0.945**	0.939	0.897	**0.909**
GM	**0.907**	0.900	0.864	**0.876**
LV	0.964	0.959	0.931	**0.948**
Tha	0.953	0.951	0.930	**0.939**
HC	0.909	0.907	0.884	**0.893**
CdN	0.932	0.930	0.907	**0.921**
Pu	0.934	0.931	0.906	**0.918**
GP	0.914	0.911	0.874	**0.905**
ALL	**0.932**	0.929	0.899	**0.914**

*Highlighted results indicate that median values are larger (P50: Wilcoxon, p < 0.05)*.

#### 4.1.1. OASIS

The models achieve high Dice scores and low variability. *Se* and *Pr* are very similar for CNN_OASIS_ and CNN_OASIS-DA_ (min: *Se*_*GM*_ = 0.87, *Pr*_*GM*_ = 0.87; mean: $\bar{Se}=0.94$, $\bar{Pr}=0.94$). There is no statistical difference between the Dice score results (Wilcoxon: *p* > 0.05, Levene: *p* > 0.05), except for WM and GM, where CNN_OASIS_ tends to perform better (Wilcoxon, *p* < 0.05). Although statistically different, the difference is marginal, especially when considering the lower limits of the distributions, as can be appreciated on the left hand side panel of Figure 3.

#### 4.1.2. MS Dataset

CNN_OASIS-DA_ outperforms CNN_OASIS_ for all structures (Wilcoxon: *p* ≪ 0.05). *Se* values are also lower in the CNN_OASIS_ model (min: *Se*_*GP*_ = 0.81, mean: $\bar{Se}=0.88$), while *Pr* values are overall comparable between the two models, with local differences for specific tissues (refer to Supplementary Table 1 for details). Additionally, we can observe in the right hand side panel of Figure 3 that the variability and incidence of outliers is reduced for CNN_OASIS-DA_. All these observations imply that the addition of GMM-DA greatly improves the performance of the model to new data containing unseen scanner types from different centers.

### 4.2. GMM Augmentation of a Heterogeneous Dataset

**Figure 4:**
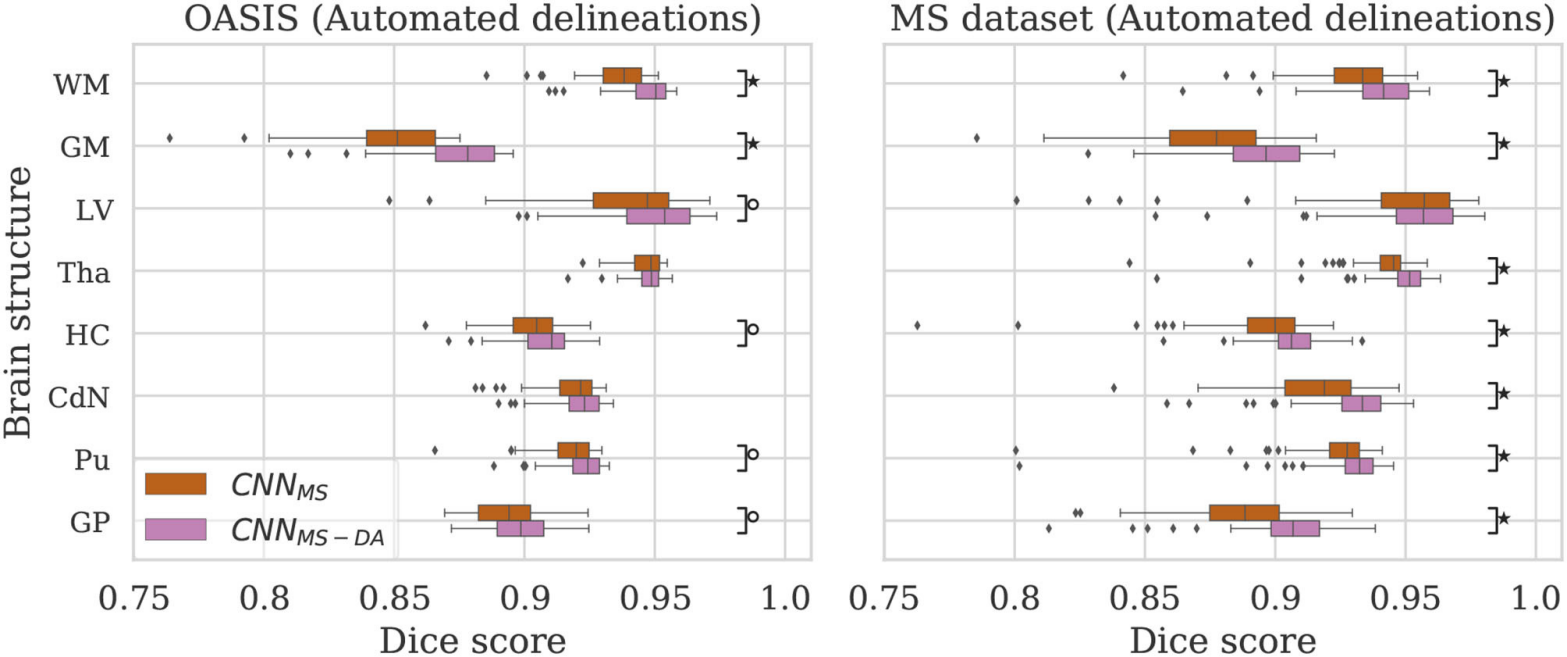
Dice scores for the CNN_MS_ and CNN_MS-DA_ models on the OASIS (left) and MS dataset (right) test sets. Marks indicate that there is a significant difference between the two models (Wilcoxon, °*p* < 0.05, ^⋆^*p* < 5×10^−4^).

Now that we have established that the addition of GMM-DA is beneficial for the generalization of a model trained on a homogeneous dataset to multi-scanner settings, we evaluate the performance of a model trained on the MS dataset, which is very heterogeneous. We additionally investigate the effect of adding GMM-DA when training on a dataset with these characteristics. The CNN_MS_ and CNN_MS-DA_ models are evaluated in the same way as the above, and results are summarized in Figure 4 and Table 3. The corresponding *Se* and *Pr* results can be found in the Supplementary Table 2.

**Table 3:** Summary of the Dice score (DC) performance of models trained on the MS dataset (CNN_MS_ and CNN_MS-DA_) and tested on the OASIS and MS dataset test sets.

	**OASIS-test set**		**MS dataset-test set**	
**Tissues**	**CNN_MS_**	**CNN_MS-DA_**	**CNN_MS_**	**CNN_MS-DA_**
WM	0.938	**0.950**	0.934	**0.942**
GM	0.851	**0.878**	0.877	**0.896**
LV	0.947	**0.954**	0.957	0.957
Tha	0.949	0.949	0.945	**0.952**
HC	0.905	**0.910**	0.900	**0.906**
CdN	0.922	0.923	0.919	**0.933**
Pu	0.920	**0.924**	0.928	**0.932**
GP	0.894	**0.899**	0.888	**0.907**
ALL	0.916	**0.923**	0.919	**0.928**

*Highlighted results indicate that median values are larger (P50: Wilcoxon, p < 0.05)*.

#### 4.2.1. OASIS

The MS dataset does not contain images with the same characteristics as OASIS. This explains a drop in performance in terms of DC for CNN_MS_ on the OASIS test set. From Table 3 we can observe that after the addition of GMM-DA the performance increases: CNN_MS-DA_ performs better for all the structures, with the exception of Tha and CdN, where there is no statistical difference in terms of performance (see the left hand side panel of Figure 4).

#### 4.2.2. MS Dataset

As mentioned in section 3, the MS test set contains scanner types which were not present in the training set. CNN_MS-DA_ outperforms CNN_MS_ for all structures (Wilcoxon: *p* ≪ 0.05) except LV (Wilcoxon: *p* > 0.05) in terms of DC (see Table 3 and the right hand side panel of Figure 4). *Se* and *Pr* values are also generally lower in the CNN_MS_ model, with local differences for specific tissues (see Supplementary Table 2 for details). This indicates that adding GMM-DA to an already heterogeneous dataset can further increase the generalizability of the network.

### 4.3. Comparison Between the Different Models

**Figure 5:**
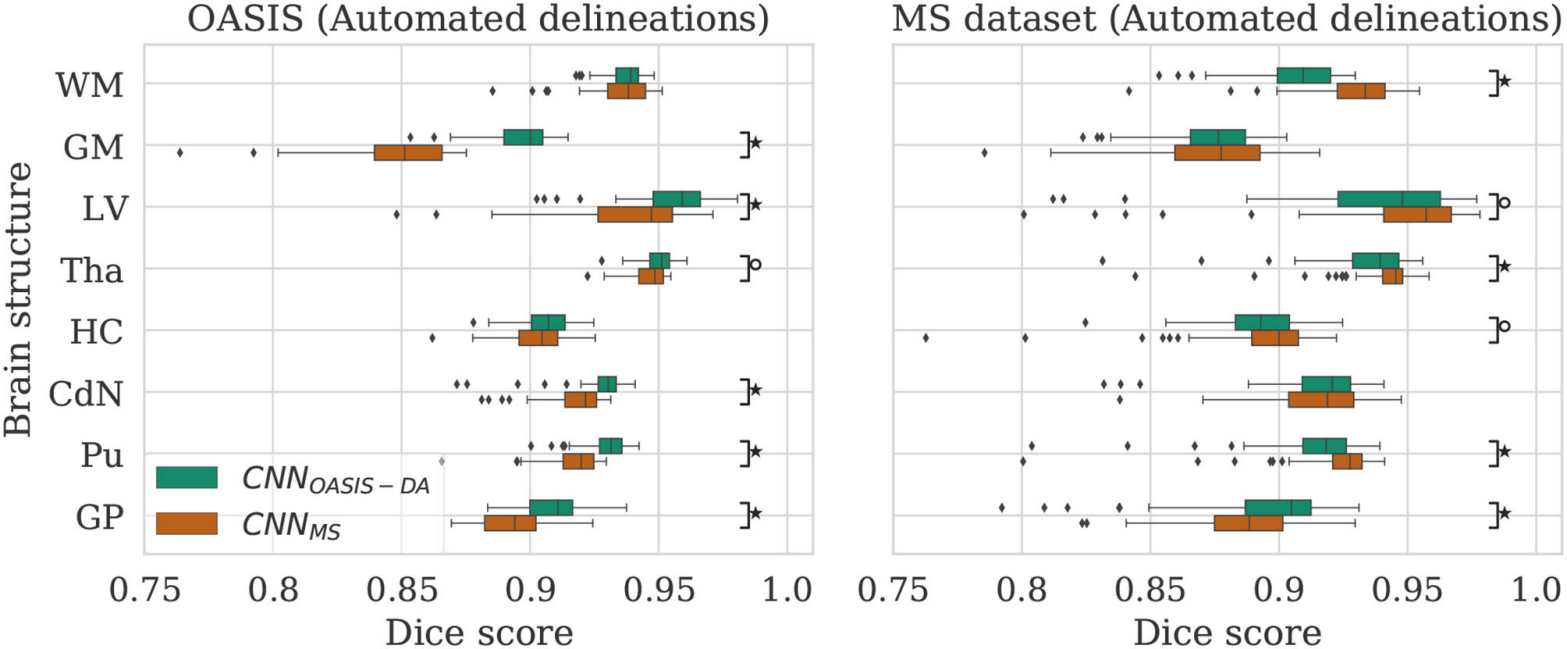
Dice scores for the CNN_OASIS-DA_ and CNN_MS_ models on the OASIS (left) and MS dataset (right) test sets. Marks indicate that there is a significant difference between the two models (Wilcoxon, °*p* < 0.05, ^⋆^*p* < 5×10^−4^).

Given that large multi-scanner and multi-center datasets are not commonly available to researchers, we are particularly interested in the comparison between the model trained on OASIS with augmentation (CNN_OASIS-DA_) against the model trained on the MS dataset without augmentation (CNN_MS_). To facilitate the comparison, the performance of both models is displayed in Figure 5.

#### 4.3.1. OASIS

For most of the evaluated structures, CNN_MS_ shows a significant decrease in performance in comparison to CNN_OASIS-DA_. It is expected that the models trained on the MS dataset have generally lower performance than the models trained on OASIS, since the images in the MS dataset training set do not share the same characteristics as the ones in OASIS (as previously illustrated in Figure 2). The addition of GMM-DA to CNN_MS_ can help reduce this performance gap, as seen in the previous section.

#### 4.3.2. MS Dataset

Analyzing the right hand side panel of Figure 5, it is interesting to verify that CNN_OASIS-DA_ approximates the variability of the CNN_MS_ for all the structures. In terms of median DC values it sometimes equals or even surpasses its performance (GM, GP and CdN). It is important to keep in mind that the MS dataset contains pathological images which are not present in OASIS. CNN_MS_ has been exposed to many more types of images, with some patients possibly presenting a small number of lesions. However, the contrary is not true, given that OASIS only contains images from healthy subjects. At best, the networks trained on this data were exposed to a few lesions present in the older subjects' scans. It is thus not possible to guarantee that the differences in performance between CNN_MS_ and CNN_OASIS-DA_ on a pathological dataset are caused only by scanner or acquisition variability. Nevertheless, these results show that with a simple data augmentation strategy it is possible to achieve competitive results on unseen data from various scanners and centers.

**Figure 6:**
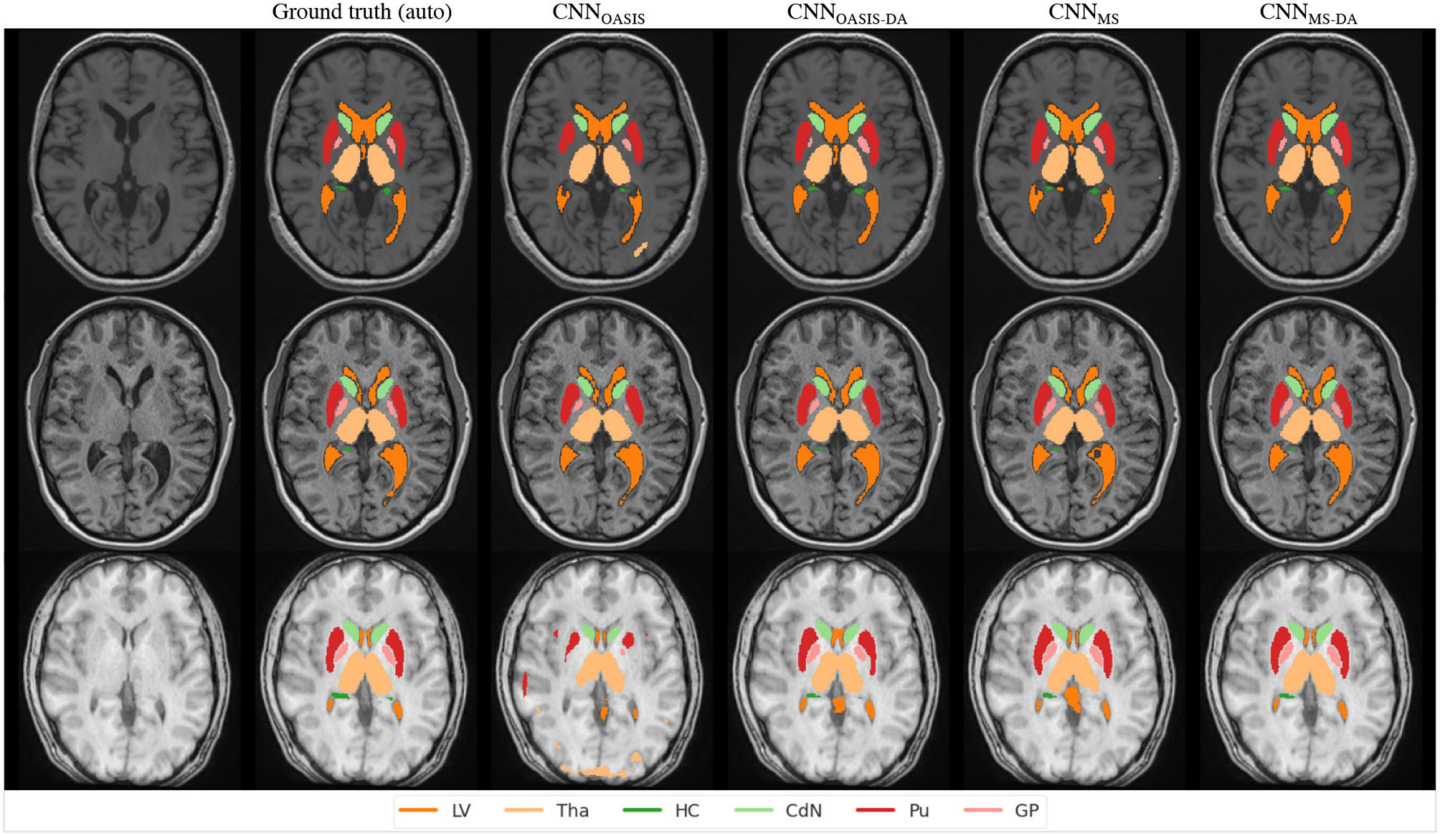
Segmentation results for the different models on three test examples from the MS dataset. The automated ground truth is computed by ico**brain**. Results from CNN_OASIS_ are very variable depending on image intensity. The addition of GMM-DA improves the segmentation prediction.

In order to visualize the different results, Figure 6 illustrates the results obtained on three different images from the MS dataset using the four different models described so far. For simplicity, WM and GM are not shown. Looking at this figure it is very clear that when the image contrast is not good, the CNN_OASIS_ model can produce segmentation results which infiltrate WM and CGM regions in unexpected ways. The addition of GMM-DA brings the results much closer to the ground truth results.

### 4.4. Evaluation on Manual Labels

**Figure 7:**
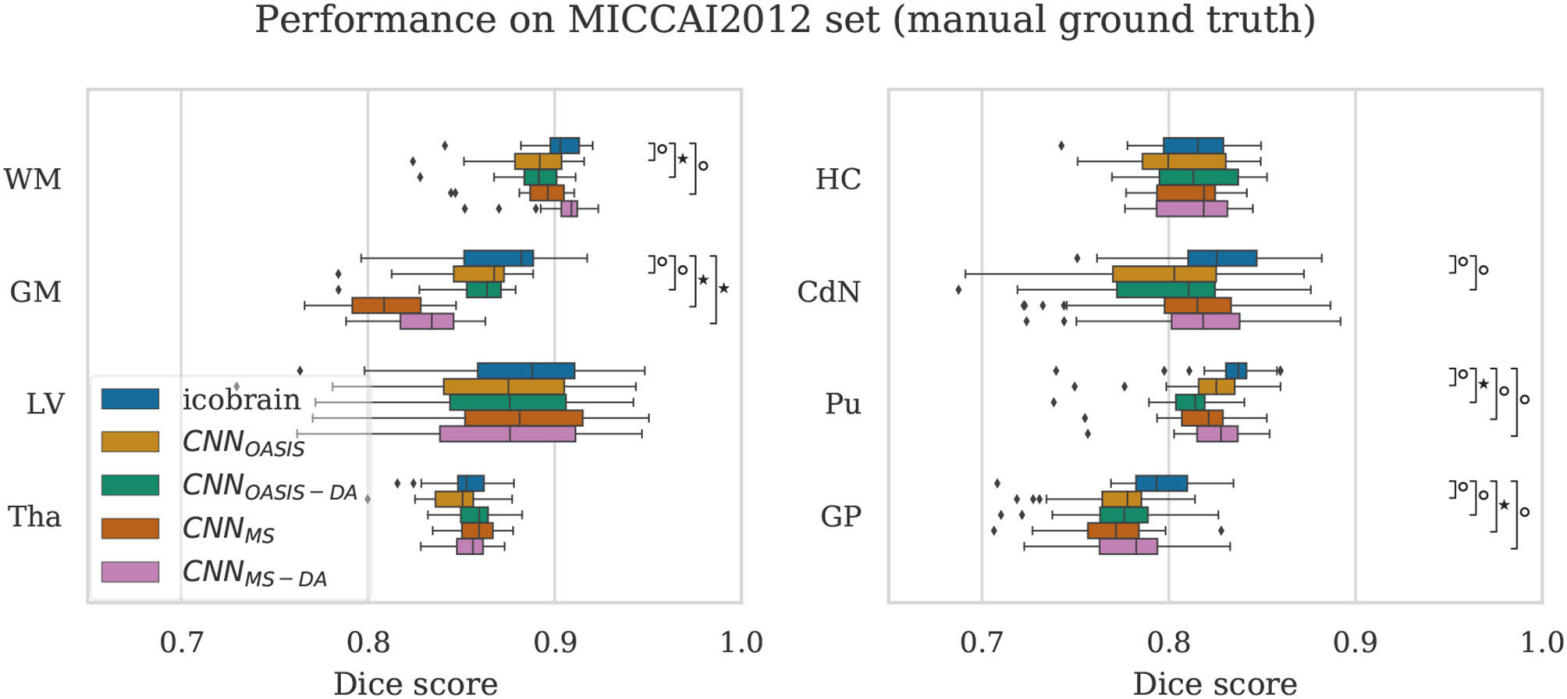
Dice scores on the MICCAI2012 test set for ico**brain**, CNN_OASIS_, CNN_OASIS-DA_, CNN_MS_ and CNN_MS−DA_ models. Asterisks indicate that there is a significant difference between the result and ico**brain** (Wilcoxon, °*p* < 0.05, ^⋆^*p* < 5×10^−4^).

To validate the performance of the models on manual segmentations we evaluate them on the MICCAI 2012 dataset. It is interesting to compare their performance against the performance of the method used to get the automated labels the models were trained on (ico**brain**). The results are summarized in Figure 7, where results which are statistically different to ico**brain** are indicated (Wilcoxon: *p* < 0.05).

For most structures the models reach comparable performance. CNN_MS−DA_ is the model with overall best performance, but still does not surpass ico**brain**. For GM, CNN_MS_ and CNN_MS−DA_ achieve much lower performance than the other models. This is in line with the results observed for the OASIS dataset. Recalling that this dataset is derived from a subset of OASIS, CNN_OASIS_ and CNN_OASIS-DA_ were exposed to images with these characteristics during training, while CNN_MS_ and CNN_MS−DA_ were not. Variances are not statistically different for any tissue type. *Se* and *Pr* values are also comparable for all models, with mean $\bar{Se}\approx 0.84,\bar{Pr}\approx 0.85$.

### 4.5. Consistency on *Test-Retest* Data

By evaluating the models on the test-retest dataset described in section 3 it is possible to evaluate how each model deals with differences in scanner type. As previously mentioned, the dataset contains two repetitions per scanner in two or three different scanners. We compute the difference in predicted volume for each of the evaluated structures between same scanner repetitions (*intra-scanner* differences) and between the repetitions in different scanners (*inter-scanner* differences). We consider all possible scanner combinations, which means that we end up with 26 intra-scanner and 88 inter-scanner repetitions. We compare the performance of our methods against ico**brain**. As already mentioned, this method is clinically available. However, when performing longitudinal evaluations, this method has a key limitation: the results are considered reliable only if the two images being analyzed were acquired in the same, or compatible, scanner. As such, we are interested in achieving better inter-scanner volume estimation differences, and we consider inter-scanner results to be consistent if the volume differences are in a comparable range to the intra-scanner differences obtained by ico**brain**.

**Figure 8:**
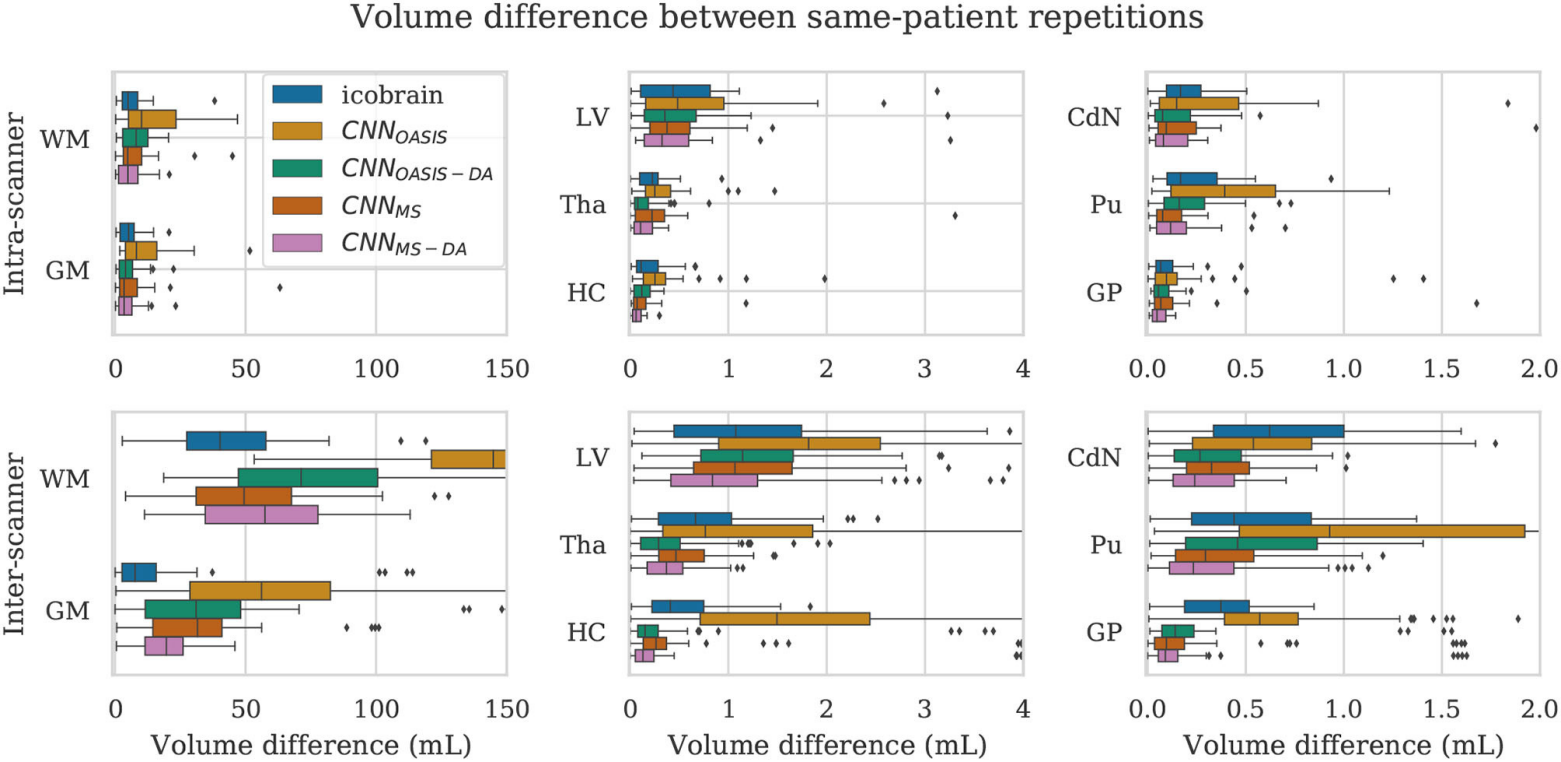
Volume difference between intra-scanner and inter-scanner repetitions from the same patient. To be able to keep the same scale for intra-scanner and inter-scanner the CNN_OASIS_ results are sometimes not fully shown.

For a simplified overview of the results, we plot the distribution of volume differences for all the considered brain structures in Figure 8. Additionally, in Table 4 we showcase the results in terms of median and *P*90, which translates the variability in the distributions. We exclude the CNN_OASIS_ model from the table, since it is clear from Figure 8 and Table 2 that the performance of this method is low for multi-scanner datasets.

**Table 4:** Volume differences (mL) between intra- and inter-scanner repetitions from the same patient.

**Intra-scanner differences**									
		**WM**	**GM**	**LV**	**Tha**	**HC**	**CdN**	**Pu**	**GP**
	P50	5.00	5.22	0.44	0.23	0.11	0.17	0.17	0.07
ico**brain**	P90	**12.94**	12.38	1.04	0.47	0.56	0.43	0.48	0.19
	P50	8.08	4.06	0.35	**0.08**	0.12	**0.08**	0.16	0.06
CNN_OASIS-DA_	P90	19.72	13.54	1.17	0.43	0.31	0.34	0.44	0.16
	P50	**4.86**	**3.48**	0.38	0.22	0.07	0.09	**0.08**	0.07
CNN_MS_	P90	15.82	14.73	1.02	0.46	0.28	0.36	**0.28**	0.19
	P50	4.94	3.51	**0.33**	0.11	**0.06**	**0.08**	0.12	**0.05**
CNN_MS-DA_	P90	14.11	**12.12**	**0.82**	**0.33**	**0.16**	**0.26**	0.34	**0.13**
**Inter-scanner differences**									
		**WM**	**GM**	**LV**	**Tha**	**HC**	**CdN**	**Pu**	**GP**
	P50	**40.26**	**7.68**	1.08	0.67	0.41	0.62	0.44	0.37
ico**brain**	P90	**76.52**	**23.77**	2.73	1.38	1.15	1.16	1.04	0.67
	P50	71.28	31.07	1.15	**0.29**	0.15	0.27	0.46	0.14
CNN_OASIS-DA_	P90	117.65	65.34	3.15	1.21	0.56	0.82	1.11	0.30
	P50	49.43	31.58	1.07	0.47	0.26	0.33	0.29	0.10
CNN_MS_	P90	94.36	55.98	**2.56**	1.46	0.65	0.91	1.13	0.73
	P50	57.45	19.71	**0.84**	0.37	**0.13**	**0.24**	**0.23**	**0.09**
CNN_MS-DA_	P90	93.47	35.75	2.85	**0.88**	**0.29**	**0.57**	**0.87**	**0.27**

*Best results are highlighted*.

Globally we observe that intra-scanner differences are much lower than inter-scanner differences for all the models. In the intra-scanner case, CNN_OASIS_ produces a higher error than the other models for all structures. Interestingly, CNN_OASIS-DA_ produces very stable results, comparable to or even better than ico**brain** for several structure types (Tha, HC, CdN). CNN_MS-DA_ produces the most consistent results for most of the structures, especially when considering *P*90.

Regarding inter-scanner differences, we observe that the CNN_OASIS_ model produces extremely large variability. The other models either compare to ico**brain** or produce more consistent results. The exception is WM and GM, where ico**brain** still outperforms the other methods in terms of consistency. This is in line with the previous observations that performance (in terms of Dice) was lower in these two tissues. The most important observation is that CNN_MS-DA_ produces the most consistent results for all the substructures. The results for this model are sometimes comparable to the values obtained by ico**brain** in the intra-scanner case (noticeably for HC and GP). Overall, the addition of GMM-DA results in a very significant improvement, both in comparison to ico**brain** and to the CNN_MS_ method. Additionally, a very interesting observation is that CNN_OASIS-DA_ achieves a performance which is comparable to that of CNN_MS_, sometimes even surpassing it (Tha, HC, CdN).

### 4.6. Influence of Bias Field

A bias field is an undesirable spatially smoothly varying low frequency signal that often corrupts MRI images (Juntu et al., 2005). A number of methods have been proposed to remove this signal from the images, and bias-field correction is often used as a pre-processing step. Given that this is a slow procedure which can sometimes produce underlying errors, it has become popular to skip bias field correction when using deep learning approaches, and instead allow the networks to learn the bias-field mechanisms, with good results (Kamnitsas et al., 2017).

**Figure 9:**
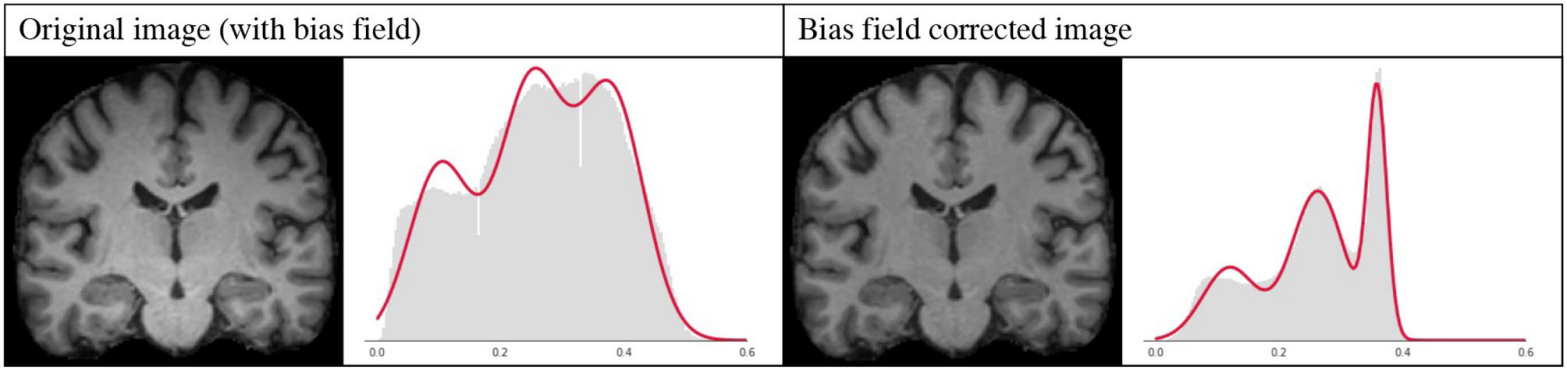
Influence of bias field on the GMM. Images and corresponding histograms with GMM prediction overlaid in red. Left: original image with bias field; right: bias field corrected image.

However, bias field correction is extremely important for GMM-based methods, since it changes the intensity profiles of the different tissues. This effect is illustrated in Figure 9, where the histogram of a bias field corrected image is compared to that of an image with bias field. It is very likely that when applying GMM-DA some of the voxels corresponding to WM will be treated as GM, or vice-versa. This implies that the structural information can be lost, which will very likely result in drop in the performance of a model trained on images with bias field and the addition of GMM-DA.

To test our hypothesis we trained two models, following the same scheme as in the previous experiments, on images with bias field. More specifically, we repeated the experiment from the previous section training on the MS dataset without the bias field correction at pre-processing. The results of this experiment are detailed in Table 5. As expected, applying GMM-DA on this type of data either decreases the performance of the method (WM, GM and LV), or has no effect on the segmentation performance. This is in line with our hypothesis and indicates that the GMM-DA should be applied on bias-field corrected images.

**Table 5:** Summary of the performance of models trained and evaluated on data with bias field on MS dataset.

	**MS dataset-test set (with bias field)**			
	**CNN_MS-BF_**		**CNN_MS-BF-DA_**	
**Tissue**	**DC(P50)**	**DC(P10)**	**DC(P50)**	**DC(P10)**
WM	**0.950**	0.920	0.945	0.914
GM	**0.914**	0.877	0.903	0.859
LV	**0.941**	**0.854**	0.845	0.554
Tha	0.937	0.911	0.937	0.896
HC	0.865	0.842	0.858	0.827
CdN	0.915	0.880	0.910	0.865
Pu	0.903	0.885	0.911	0.891
GP	0.855	0.803	0.854	0.807
ALL	0.910	0.872	0.895	0.827

*DC, Dice scores. Highlighted results indicate that median values are larger (P50: Wilcoxon, p < 0.05) or variances are lower (P10: Levene, p < 0.05)*.

## 5. Conclusions and Future Work

In this work we present a novel intensity-based data augmentation strategy. The main goal of this approach is to aid models trained on scanner- and center-homogeneous datasets generalizing to multi-scanner, multi-center data. The proposed method is fast, simple and can be added to any MRI training pipeline to generate images on-the-fly. We observed that applying the augmentation step while training on homogeneous data leads to a pronounced improvement in performance when the trained model is tested in multi-scanner data from difference centers. This is the case in terms of segmentation quality (as measured by Dice score), but also in the consistency of the produced prediction (as measured in terms of volume differences). When applied to the test-retest dataset there is a remarkable improvement, especially for repetitions in different scanners. The baseline model trained on homogeneous data produces extremely inconsistent results, while the same model with addition of GMM-DA compares to a model trained on multi-scanner, multi-center data. We additionally verify that applying GMM-DA when training a model on multi-center data results in an increase in performance, again both in terms of accuracy and consistency of the predictions. These observations are particularly interesting because large multi-scanner, multi-center datasets are not commonly available to researchers in the field. Nevertheless, even when such a dataset is available, it is possible to obtain even more generalization by adding a simple augmentation strategy.

It should be noted that the heterogeneous dataset contains several sources of variability, including acquisition sequence parameters. The resulting contrast variability is also addressed by the GMM-DA. Therefore, we can attribute the improvement in the generalization capabilities of the CNN not only to scanner, but also to generalization to unseen acquisition parameters, or other center-specific factors.

It is possible that combining this method with other DA procedures would result in an even more robust model. Nevertheless, we opted to restrict the augmentation procedures such that we could observe the added value of our method alone. Additionally, since the images were registered to MNI space adding geometric transformations such as rotations and flips is not necessary. Nonetheless, it is expected that the DA algorithm still works well if the images are in native space. Registration was performed as a way to simplify the learning of the network, since we were interested in comparing the effect of the augmentation step in a simplified setting.

There are a few limitations to the present work. Namely, the images need to be bias-field corrected as a pre-processing step to successfully apply the GMM-DA. We don't see this as a disadvantage, since GMM-DA is only needed at training time. We argue that it would be possible to add back the bias-field to the augmented image, which would allow the model to be effectively trained with bias field. This step would allow the final trained model to generalize to images with bias-field, thus eliminating the need for bias-field correction at inference time. Experimental validation of this claim remains out of the scope of the present work, given that it is related to improving the overall model performance, and is not connected to the effectiveness of the proposed approach.

Additionally, the presence of pathology in the MS dataset introduces an extra source of variability. In images with WM lesions, as is the case for MS, it is tempting to assume that a fourth component to the GMM would be a good way to capture the lesion class. However, lesions in T1w images overlap with the GM class in terms of intensity, for which reason it would be impossible to perfectly disentangle the two classes with the current framework. A more sophisticated approach would be necessary for this, likely at the cost of the possibility to generate images on-the-fly, unless lesion masks are available.

Finally, due to scarcity of manual delineations, the models were trained on automated segmentations. This is not ideal, because our model is likely to inherit any bias or known problems that might exist in the ground truth. However, given that we are especially interested in the effect of the augmentation we can still make a fair comparison between the approaches.

Although we focused on the task of brain structure segmentation in T1w MRI images, we believe this simple method has the potential to be used for other tasks in medical imaging that make use of MR images. As long as there are discernible, anatomically-related peaks in the intensity histograms, the method is transferable to other MR protocols and sequences. It is an open question whether the method is helpful for different tasks without further adaptations. For tasks such as lesion segmentation we hypothesize that if lesion masks are available it would be simple to adapt the method such that contrasts and intensities are locally modified within the abnormal area. We further see potential in this method to be adapted such that it offers a fast way to replace missing modalities in tasks requiring two or more MRI modalities (e.g., as often performed for brain tumor segmentation). This would expectably come at the expense of some performance power, but could allow existing pipelines to be used on incomplete data.

Given these considerations, an immediate next step would be to apply the current method to different applications (e.g., brain age or disability scores prediction from MR images) and verify our claim. A second step would be to extend the method to different types of brain lesions when such masks are available, to model the intensity of the tissues of interest individually, and test the added value of the extended method to applications such as detection, classification and segmentation of MS lesions, stroke, or brain tumors. Additional future directions include extending the augmentation method by introducing changes to the different components of the mixture such that they are not necessarily represented by Gaussian distributions. Moreover, it would be interesting to investigate how the addition of (preferably Rician) noise to the images would impact performance on unseen scanner types. Typical geometric distortions and bias fields can also be modeled and included in a more complex data augmentation scheme.

## Data Availability Statement

This research study was conducted using human subject data partly made available in open access by OASIS (https://www.oasis-brains.org/) (Marcus et al., 2007) and manual labelings by Neuromorphometrics, Inc. (http://Neuromorphometrics.com/) under academic subscription (Landman and Warfield, 2012). The data is released under the Creative Commons Attribution NonCommercial license (CC BY-NC) with no end date. The MS dataset is a subset of data processed with icobrain ms in clinical practice, for which subjects had agreed to allow icometrix to use an anonymized version of the already analysed MR images for research purposes.

## Author Contributions

MM: conceptualization, investigation, methodology, software, and writing—original draft preparation. ER and RP: methodology and writing—reviewing and editing. NP: conceptualization and writing—reviewing and editing. KV: supervision, writing—reviewing and editing, and funding acquisition. DS: supervision, conceptualization, writing—reviewing and editing, and funding acquisition. All authors contributed to the article and approved the submitted version.

## Conflict of Interest

MM, ER, NP, RP, and DS are employed by ico**metrix**. The remaining author declares that the research was conducted in the absence of any commercial or financial relationships that could be construed as a potential conflict of interest.

## Publisher's Note

All claims expressed in this article are solely those of the authors and do not necessarily represent those of their affiliated organizations, or those of the publisher, the editors and the reviewers. Any product that may be evaluated in this article, or claim that may be made by its manufacturer, is not guaranteed or endorsed by the publisher.

## References

[B1] Ashburner J. Friston K. J. (2005). Unified segmentation. Neuroimage 26, 839–851. 10.1016/j.neuroimage.2005.02.018 15955494

[B2] Bagc,i U. Udupa J. K. Bai L. (2010). The role of intensity standardization in medical image registration. Pattern Recognit. Lett. 31, 315–323. 10.1016/j.patrec.2009.09.010

[B3] Billot B. Greve D. N. Van Leemput K. Fischl B. Iglesias J. E. Dalca A. (2020). A learning strategy for contrast-agnostic mri segmentation, in Proceedings of the Third Conference on Medical Imaging with Deep Learning, Vol. 121 of Proceedings of Machine Learning Research, (Montreal, QC: PMLR), 75–93.

[B4] Çiçek Ö. Abdulkadir A. Lienkamp S. S. Brox T. Ronneberger O. (2016). 3D U-Net: learning dense volumetric segmentation from sparse annotation, in Medical Image Computing and Computer-Assisted Intervention-MICCAI 2016 (Cham: Springer International Publishing), 424–432.

[B5] Dempster A. P. Laird N. M. Rubin D. B. (1977). Maximum likelihood from incomplete data via the em algorithm. J. R. Stat. Soc. Ser. B 39, 1–38. 10.1111/j.2517-6161.1977.tb01600.x

[B6] Dewey B. E. Zhao C. Reinhold J. C. Carass A. Fitzgerald K. C. Sotirchos E. S.. (2019). DeepHarmony: a deep learning approach to contrast harmonization across scanner changes. Magn. Reson. Imaging 64m 160–170. 10.1016/j.mri.2019.05.041 31301354 PMC6874910

[B7] Fortin J.-P. Cullen N. Sheline Y. I. Taylor W. D. Aselcioglu I. Cook P. A.. (2018). Harmonization of cortical thickness measurements across scanners and sites. Neuroimage 167, 104–120. 10.1016/j.neuroimage.2017.11.024 29155184 PMC5845848

[B8] Fortin J.-P. Sweeney E. M. Muschelli J. Crainiceanu C. M. Shinohara R. T. (2016). Removing inter-subject technical variability in magnetic resonance imaging studies. Neuroimage 132, 198–212. 10.1016/j.neuroimage.2016.02.036 26923370 PMC5540379

[B9] Garcia-Dias R. Scarpazza C. Baecker L. Vieira S. Pinaya W. H. Corvin A.. (2020). Neuroharmony: A new tool for harmonizing volumetric MRI data from unseen scanners. Neuroimage 220, 117127. 10.1016/j.neuroimage.2020.117127 32634595 PMC7573655

[B10] Giorgio A. De Stefano N. (2013). Clinical use of brain volumetry. J. Magn. Reson. Imaging 37, 1–14. 10.1002/jmri.23671 23255412

[B11] Isensee F. Jaeger P. F. Kohl S. A. Petersen J. Maier-Hein K. H. (2021). nnU-Net: a self-configuring method for deep learning-based biomedical image segmentation. Nat. Methods 18, 203–211. 10.1038/s41592-020-01008-z 33288961

[B12] Jain S. Sima D. M. Ribbens A. Cambron M. Maertens A. Van Hecke W.. (2015). Automatic segmentation and volumetry of multiple sclerosis brain lesions from MR images. NeuroImage Clin. 8:367–375. 10.1016/j.nicl.2015.05.003 26106562 PMC4474324

[B13] Jog A. Hoopes A. Greve D. N. Van Leemput K. Fischl B. (2019). PSACNN: Pulse sequence adaptive fast whole brain segmentation. Neuroimage 199, 553–569. 10.1016/j.neuroimage.2019.05.033 31129303 PMC6688920

[B14] Juntu J. Sijbers J. Van Dyck D. Gielen J. (2005). Bias field correction for mri images, in Computer Recognition Systems (Berlin; Heidelberg: Springer Berlin Heidelberg), 543–551.

[B15] Kamnitsas K. Ledig C. Newcombe V. F. Simpson J. P. Kane A. D. Menon D. K.. (2017). Efficient multi-scale 3D CNN with fully connected CRF for accurate brain lesion segmentation. Med. Image Anal. 36:61–78. 10.1016/j.media.2016.10.004 27865153

[B16] Krizhevsky A. Sutskever I. Hinton G. E. (2012). Imagenet classification with deep convolutional neural networks, in Advances in Neural Information Processing Systems, Vol. 25. (Lake Tahoe, NV: Curran Associates, Inc).

[B17] Landman B. Warfield S. (2012). Miccai 2012 workshop on multi-atlas labeling, in MICCAI Grand Challenge and Workshop on Multi-Atlas Labeling (Nice: CreateSpace Independent Publishing Platform).

[B18] Mårtensson G. Ferreira D. Granberg T. Cavallin L. Oppedal K. Padovani A.. (2020). The reliability of a deep learning model in clinical out-of-distribution MRI data: a multicohort study. Med. Image Anal. 66:101714. 10.1016/j.media.2020.101714 33007638

[B19] Maas A. L. Hannun A. Y. Ng A. Y. (2013). Rectifier Nonlinearities Improve Neural Network Acoustic Models. Technical report.

[B20] Marcus D. S. Wang T. H. Parker J. Csernansky J. G. Morris J. C. Buckner R. L. (2007). Open Access Series of Imaging Studies (OASIS): Cross-sectional MRI data in young, middle aged, nondemented, and demented older adults. J. Cogn. Neurosci. 19, 1498–1507. 10.1162/jocn.2007.19.9.1498 17714011

[B21] Meyer M. I. de la Rosa E. Barros N. Paolella R. Van Leemput K. Sima D. M. (2021). An augmentation strategy to mimic multi-scanner variability in MRI, in 2021 IEEE 18th IEEE International Symposium on Biomedical Imaging (Nice). 1196–1200.

[B22] Meyer M. I. de la Rosa E. Van Leemput K. Sima D. M. (2019). Relevance vector machines for harmonization of MRI brain volumes using image descriptors, in Lecture Notes in Computer Science (Including Subseries Lecture Notes in Artificial Intelligence (LNAI) and Lecture Notes Bioinformatics), Vol. 11796 LNCS (Shenzhen), 77–85.

[B23] Milletari F. Navab N. Ahmadi S.-A. (2016). V-net: Fully convolutional neural networks for volumetric medical image segmentation, in 2016 Fourth International Conference on 3D Vision (3DV) (Stanford University, CA), 565–571.

[B24] Moyer D. Golland P. (2021). Harmonization and the worst scanner syndrome. arXiv, cs.LG/2101.06255.

[B25] Nyúl L. G. Udupa J. K. (1999). On standardizing the MR image intensity scale. Magn. Reson. Med. 42, 1072–1081. 10571928 10.1002/(sici)1522-2594(199912)42:6<1072::aid-mrm11>3.0.co;2-m

[B26] Ourselin S. Roche A. Subsol G. Pennec X. Ayache N. (2001). Reconstructing a 3d structure from serial histological sections. Image Vis. Comput. 19, 25–31. 10.1016/S0262-8856(00)00052-4

[B27] Pedregosa F. Varoquaux G. Gramfort A. Michel V. Thirion B. Grisel O.. (2011). Scikit-learn: machine learning in Python. J. Mach. Learn. Res. 12, 2825–2830. Available online at: http://jmlr.org/papers/v12/pedregosa11a.html

[B28] Ronneberger O. Fischer P. Brox T. (2015). U-net: Convolutional networks for biomedical image segmentation, in Medical Image Computing and Computer-Assisted Intervention-MICCAI 2015 (Cham: Springer International Publishing), 234–241.

[B29] Salimans T. Kingma D. P. (2016). Weight normalization: a simple reparameterization to accelerate training of deep neural networks, in Conference on Neural Information Processing Systems (Barcelona), 901–909.

[B30] Shin H.-C. Tenenholtz N. A. Rogers J. K. Schwarz C. G. Senjem M. L. Gunter J. L.. (2018). Medical image synthesis for data augmentation and anonymization using generative adversarial networks, in Simulation and Synthesis in Medical Imaging (Cham: Springer International Publishing), 1–11.

[B31] Shinohara R. T. Sweeney E. M. Goldsmith J. Shiee N. Mateen F. J. Calabresi P. A.. (2014). Statistical normalization techniques for magnetic resonance imaging. Neuroimage Clin. 6, 9–19. 10.1016/j.nicl.2014.08.008 25379412 PMC4215426

[B32] Sietsma J. Dow R. J. (1991). Creating artificial neural networks that generalize. Neural Networks 4, 67–79. 10.1016/0893-6080(91)90033-2

[B33] Simard P. Steinkraus D. Platt J. (2003). Best practices for convolutional neural networks applied to visual document analysis, in Seventh International Conference on Document Analysis and Recognition, 2003. Proceedings (Edinburgh), 958–963.

[B34] Struyfs H. Sima D. M. Wittens M. Ribbens A. Pedrosa de Barros N. Phan T. V.. (2020). Automated MRI volumetry as a diagnostic tool for Alzheimer's disease: validation of icobrain dm. Neuroimage Clin. 26:102243. 10.1016/j.nicl.2020.102243 32193172 PMC7082216

[B35] Sudre C. H. Li W. Vercauteren T. Ourselin S. Jorge Cardoso M. (2017). Generalised dice overlap as a deep learning loss function for highly unbalanced segmentations, in Deep Learning in Medical Image Analysis and Multimodal Learning for Clinical Decision Support (Cham: Springer International Publishing), 240–248. 10.1007/978-3-319-67558-9_28 PMC7610921 34104926

[B36] Tustison N. J. Avants B. B. Cook P. A. Zheng Y. Egan A. Yushkevich P. A.. (2010). N4itk: Improved n3 bias correction. IEEE Trans Med Imaging 29, 1310–1320. 10.1109/TMI.2010.2046908 20378467 PMC3071855

[B37] Van Leemput K. Maes F. Vandermeulen D. Suetens P. (1999). Automated model-based tissue classification of MR images of the brain. IEEE Trans. Med. Imaging 18, 897–908. 10.1109/42.811270 10628949

[B38] Wang L. Lai H. M. Barker G. J. Miller D. H. Tofts P. S. (1998). Correction for variations in MRI scanner sensitivity in brain studies with histogram matching. Magn. Reson. Med. 39, 322–327. 10.1002/mrm.1910390222 9469718

[B39] Wrobel J. Martin M. L. Bakshi R. Calabresi P. A. Elliot M. Roalf D.. (2020). Intensity warping for multisite MRI harmonization. Neuroimage 223:117242. 10.1016/j.neuroimage.2020.117242 32798678 PMC8412236

[B40] Zhao A. Balakrishnan G. Durand F. Guttag J. V. Dalca A. V. (2019a). Data augmentation using learned transformations for one-shot medical image segmentation, in Proceedings of the IEEE/CVF Conference on Computer Vision and Pattern Recognition (CVPR) (Long Beach, CA).

[B41] Zhao F. Wu Z. Wang L. Lin W. Xia S. Shen D.. (2019b). Harmonization of infant cortical thickness using surface-to-surface cycle-consistent adversarial networks, in Lecture Notes in Computer Science (Including Subseries Lecture Notes in Artificial Intelligence (LNAI) and Lecture Notes Bioinformatics), Vol. 11767 LNCS, 475–483. 32128523 10.1007/978-3-030-32251-9_52 PMC7052700

[B42] Zhuge Y. Udupa J. K. (2009). Intensity standardization simplifies brain MR image segmentation. Comput. Vis. Image Understand. 113, 1095–1103. 10.1016/j.cviu.2009.06.003 20161360 PMC2777695

