# Supplementary material for: A Contrast Augmentation Approach to Improve Multi-Scanner Generalization in MRI
Source: Front Neurosci. 2021 Aug 31;15:708196. doi: 10.3389/fnins.2021.708196 (PMC8439197; doi:10.3389/fnins.2021.708196)
Supplement: Supplementary file 1 [file Data_Sheet_1.PDF]

Supplementary Material

1 SUPPLEMENTARY TABLES

Table S1. Summary of the performance on automated ground truth of models trained on the OASIS data. Se: Sensitivity, Pr: Precision. Highlighted results indicate that median values are larger ($P50$: Wilcoxon, $p < 0.05$).

	OASIS - test set				MS Dataset - test set			
	CNN_{OASIS}		$CNN_{OASIS-DA}$		CNN_{OASIS}		$CNN_{OASIS-DA}$	
Tissues	Se	Pr	Se	Pr	Se	Pr	Se	Pr
WM	0.931	**0.958**	0.933	0.948	**0.932**	0.863	0.900	**0.922**
GM	**0.879**	0.933	0.868	0.934	0.860	0.863	**0.881**	**0.875**
LV	0.968	0.967	0.965	0.957	0.893	**0.984**	**0.965**	0.944
Tha	0.963	**0.943**	**0.972**	0.933	0.935	0.924	**0.952**	0.929
HC	0.945	**0.878**	**0.955**	0.867	0.872	**0.903**	**0.943**	0.851
CdN	0.944	**0.923**	**0.954**	0.907	0.871	**0.955**	**0.920**	0.925
Pu	0.950	0.920	0.948	0.917	0.896	**0.917**	**0.940**	0.903
GP	0.944	**0.887**	**0.957**	0.865	0.813	**0.936**	**0.912**	0.894
ALL	0.941	**0.926**	**0.944**	0.916	0.884	**0.91**	**0.927**	0.906

Table S2. Summary of the performance on automated ground truth of models trained on the MS dataset. Se: Sensitivity, Pr: Precision. Highlighted results indicate that median values are larger ($P50$: Wilcoxon, $p < 0.05$).

	OASIS - test set				MS Dataset - test set			
	CNN_{MS}		CNN_{MS-DA}		CNN_{MS}		CNN_{MS-DA}	
Tissues	Se	Pr	Se	Pr	Se	Pr	Se	Pr
WM	0.905	**0.980**	**0.945**	0.959	0.923	0.947	**0.934**	**0.953**
GM	0.801	0.913	**0.828**	**0.935**	0.839	0.921	**0.872**	0.927
LV	**0.984**	0.916	0.967	**0.944**	0.956	**0.964**	**0.965**	0.959
Tha	0.962	0.940	0.958	0.941	**0.972**	0.921	0.959	**0.945**
HC	0.943	0.869	0.942	**0.882**	0.929	0.874	**0.950**	0.868
CdN	0.946	0.898	0.950	0.901	**0.958**	0.887	0.946	**0.925**
Pu	0.913	**0.925**	**0.930**	0.918	0.944	0.910	**0.955**	0.912
GP	0.956	0.840	0.952	0.849	**0.968**	0.824	0.959	**0.865**
ALL	0.926	0.910	**0.934**	**0.916**	0.936	0.906	**0.943**	**0.919**
